# Acceptability of internet interventions for youth mental health in Vietnam

**DOI:** 10.1017/gmh.2016.18

**Published:** 2016-07-14

**Authors:** K. Sobowale, M. Nguyen, B. Weiss, T. T. Hai Van, L. T. Trung

**Affiliations:** 1 Department of Psychiatry, Yale University School of Medicine, New Haven, Connecticut, USA; 2 Department of Clinical Psychology, Vietnam National University, Hanoi, Vietnam; 3 Department of Psychology and Human Development, Vanderbilt University, Nashville, Tennessee, USA; 4 Danang Psychiatric Hospital, Danang City, Vietnam

**Keywords:** Digital health, internet interventions, technology, low- and middle-income countries, mental health, psychiatry, depression, youth

## Abstract

**Background.:**

Despite high levels of mental illness, Vietnamese youth have limited access to mental health care. Internet interventions, evidence-based psychotherapy treatments delivered through the internet, have the potential to increase access to mental health for youth in Vietnam. This study explored the perceptions of youths and parents toward internet interventions for youth mental health.

**Methods.:**

Four focus groups were conducted with youths (*n* = 20) and parents (*n* = 20) in Danang, Vietnam. The Technology Acceptance Model was used a framework for focus group questions. The data were analyzed using direct content analysis.

**Results.:**

Most youths and parents agreed that the internet serves well as a care delivery model. Participants expressed that the web would be useful for psychoeducation and sharing and receiving information with others. Both groups reported lack of awareness of web-based interventions and logistical concerns regarding access as main barriers. In addition, many parents were concerned about internet addiction. Specific adaptations in Vietnam such as standalone internet service centers and partnering with local organizations may benefit uptake of internet interventions.

**Conclusion.:**

This study suggests that internet-based programs for youth mental health, particularly interventions incorporating psychoeducation and social networking components, will be well received in Vietnam. Barriers need to be addressed to successfully implement internet-based treatment. Future initiatives should incorporate acceptance models to improve development of internet interventions for youth.

## Introduction

Vietnamese youth experience a significant burden of mental illness. Depression and associated risky behaviors, including alcohol abuse and suicidal behaviors are common in this group. A recent study of over 1100 high school students by Nguyen *et al*. (2013) found that 18.7% of students had depressive symptoms consistent with major depressive disorder. Similarly, a study of over 4500 youth in Hanoi found a 6-month prevalence of depression of 30–42% (Nguyen *et al*. 2012). In this study, depression was associated with drinking and smoking. Alcohol use, in turn, has been associated with suicide ideation and traffic-related accidents in young people in Vietnam (Le *et al*. 2012). The most feared consequence of depression is suicide. Unfortunately, more than 10% of Vietnamese endorse suicidal ideation, and the prevalence has increased in recent years (Huong *et al*. 2010; Phuong *et al*. 2013). Moreover, in a study of study of 1226 adolescents, 5.8% reported attempted suicide in the past 12 months (Thai, 2010).

Despite these alarming levels of mental illness, Vietnamese adolescents and young adults have limited sources of help for mental illness. In Vietnam, there are 1.01 psychiatrists per 100 000 people (WHO, 2011), compared to an average of 15.6 in OECD countries. Even with general doctors included, the number of mental health providers is low (Doan, 2011). Child and adolescent mental health providers are even scarcer in Vietnam (Dang & Weiss, 2012). Access is also limited by geographic distribution of providers as psychiatrists are stationed at psychiatric hospitals, where mental health care is centered. The mental health system is not well integrated into other settings. Recently, the Vietnam Ministry of Health began expanding community outreach through primary care facilities to reach those afflicted with mental illness (Ng *et al*. 2011). However, effectiveness of treatment in primary care is limited (Doan, 2011) by lack of adequate training to screen for mental disorders and shortages of psychotropic medications, the main treatment modality for mental disorders in Vietnam (Niemi *et al*. 2010). In addition, the community expansion initiative primarily focuses on schizophrenia and epilepsy, which are not the most common mental illnesses in Vietnamese youth (Ng *et al*. 2011).

Internet interventions are a novel way to address the treatment gap in young people in Vietnam. Internet interventions are highly structured psychotherapy-based treatments delivered via internet. These programs promote cognitive and behavioral skills used in evidence-based treatments [e.g. cognitive-behavioral therapy (CBT), interpersonal psychotherapy, etc.]. Internet-based interventions can be delivered with human support or fully automated without support (Titov *et al*. 2010; Berger *et al*. 2011*a*, *b*). While human support, even if minimal, improves outcomes (Baumeister *et al*. 2014), support does not need to be provided by mental health professionals, reducing the work burden for health care providers. For example, in the study by Titov and colleagues, both a layperson without clinician training and a psychiatrist assisted participants (estimated 60 min per participant) completing an online CBT intervention for depression. Both treatment groups had improved depressive symptoms compared with a control group, but there were no differences between treatment groups. In addition, internet interventions have proven effective for prevention and treatment of a wide variety of mental illnesses, including addictive disorders, eating disorders, anxiety and depression (Andersson, 2009; Andrews *et al.* 2010).

Internet interventions offer many advantages for youth in Vietnam. These interventions extend care beyond the walls of the traditional mental health facilities, allowing access to care at a distance. Users can access programs at times more convenient for their schedule as human support is often not required. Further, care can be received in a private setting such as home, thus avoiding stigma. In addition, internet interventions for mental illness are cost-effective compared with live therapy, pharmacological treatments, and no treatment (Hedman *et al*. 2012). Low-cost, wide dissemination, and the ability to access care regardless of time with little-to-no help of a provider, make internet interventions an attractive mode of care in Vietnam. Moreover, in Vietnam, internet availability has rapidly increased. Country-wide, internet penetration is 44% and over 50% in urban areas (Maierbrugger, 2013; Hookway, 2015). Youth (ages 15–24) are the vast majority of internet users (40%) (Maierbrugger, 2013).

The use of technology-based interventions requires an understanding of user perceptions. Multiple guidelines call for engagement of users early in the internet intervention development process to facilitate successful implementation (Doherty *et al*. 2010; Mohr *et al*. 2013). Indeed, studies repeatedly find that greater acceptability, the alignment of the product with user expectations and beliefs, improves the effectiveness of internet interventions by increasing uptake and adherence to treatment (Christensen & Mackinnon, 2006; Kaltenthaler *et al*. 2008). However, there is a lack of research exploring user acceptance of online interventions for mental health in low- and middle-income countries (LMICs) such as Vietnam. As there are currently no internet interventions for youth mental health in Vietnam and there is a dearth of evidence on the efficacy and effectiveness of web-based programs in LMICs (Arjadi *et al*. 2015), understanding acceptance is fundamental to optimally design and implement an intervention in this novel setting.

Therefore, we conducted focus groups with youth and parents in Vietnam to assess their acceptance of internet-delivered interventions for mental illness. Although qualitative methodology is ideal for understanding participants’ perspectives about new interventions and provides preliminary evidence to further development (Murtagh *et al*. 2007; Meissner, 2011), there are few qualitative studies of internet interventions in the literature. By using qualitative methods to inform development of internet programs for mental health for young people in Vietnam this study offers a unique contribution to the field.

## Methods

### Settings and participants

Youth and parents were recruited using flyers with a phone number to call. Flyers were placed on college campuses, high schools, and local supermarkets in Danang, Vietnam, a small city in central Vietnam. To diversify the sample, youth participants who responded to flyers were intentionally screened to balance of both genders and high school and college students. For parents, screening was conducted to ensure that only parents of youth (ages 15–24) were included, and a balance between men and women was sought. Recruitment ceased after predetermined target number of 20 youth and 20 parents was reached. A total of four focus groups, two with youth (ten participants per group) and two with parents (ten participants per group) were conducted in a private meeting room at Danang Psychiatric hospital. This study was approved by the Danang Psychiatry Hospital Institutional Review Board.

The resulting sample included 20 students and 20 parents. Students from two high schools and three universities partook in focus groups. The students’ ages ranged from 15 to 21 years old. Ten boys and ten girls were involved. For parents, there were 11 men and nine women who participated in focus groups. The parent age range was 34–47 years old.

### Data collection

Focus groups were conducted between March and September 2014. Each participant participated in two focus groups. The first focus group explored mental health literacy in regards to depression by providing a vignette of a high school student named Mai meeting DSM-IV criteria for unipolar depression. The second focus group took place 1–2 weeks after the first focus group, and focused on acceptance of internet interventions. The results of the second focus group are reported in this paper. Because of the novelty of internet interventions in Vietnam, focus groups were conducted to assess social norms about internet-delivered programs for mental health and to understand individual perceptions within a group of peers. Since mental illness is still stigmatized in Vietnam (Doan, 2011), a group format allowed for a safe space to share ideas and beliefs. In addition, since participants can converse with each other, there is less influence of the moderator (Madriz, 2000), allowing more participant input to inform intervention development.

Participants were completed written consent before the focus group began. Focus groups were moderated by a psychiatrist (author TTHV) at the hospital with previous experience conducting focus groups. A clinical psychologist assisted with the focus groups. Because participants participated in a series of two focus groups, they were familiar with the group moderator and assistant. The duration of focus group discussions ranged between 70 and 90 min.

To evaluate acceptance, focus groups questions were based on the Technology Acceptance Model (TAM) (Davis, 1989). The TAM is the most widely used model to understand technology-related behaviors such as internet or mobile device use. The model has been used for evaluating internet technology in a number of LMICs (Park *et al*. 2009) including Vietnam (Cuong *et al*. 2015; Lin *et al*. 2015). The model is based on the theory of reasoned action, a psychological theory of behavioral intentions (Fishbein & Ajzen, 1975). The components of the TAM have been shown to consistently predict behavior intention (Wang & Senecal, 2007). The TAM states intention to use technology will increase when:

1. The technology is perceived as useful – defined as ‘the degree to which a person believes that using a particular system would enhance his/her job performance’ (Davis, 1989). Perceived usefulness has been found to be predictive of attitudes to use technology (Teo *et al*. 2008). In this study, usefulness was used to investigate the perceived effectiveness of the intervention on depression.
2. The technology is easy to use – defined as ‘the degree to which a person believes that using a particular system would be free of physical and mental effort’ (Davis, 1989). The ease of use is thought to moderate how positively the user feels about the technology. In this study, ease of use was used to examine the effort required and intention to use the intervention.

The focus group questions based on the TAM include:

- What do you think about the usefulness of internet program to improve mental health?
- What would motivate you to use an internet website for mental health?
- What are the advantages of using the internet to improve mental health?
- What are the disadvantages of using the internet to improve mental health?
- Who should recommend an internet website to improve mental health?
- Where should this internet site be used?

Before asking these questions, participants were reminded and read the vignette of a high school student named Mai meeting DSM-IV criteria for unipolar depression, which was discussed in the first focus group. After reading the vignette, participants were informed that the topic of the second focus group was the use of an internet-based program to improve mental health for young people like Mai.

### Data analysis

The focus groups were recorded with participant's permission and subsequently transcribed and translated from Vietnamese to English. The transcriptions were analyzed using direct content analysis (Hsieh & Shannon, 2005). Specifically, a deductive coding process using predetermined codes based an existing theory, the TAM, was used for initial coding schemes. Data that were not consistent with predetermined codes were categorized under new codes or as a subcategory of existing codes. Two coders (KS and MN) independently reviewed the transcripts. Notably, MN was born in Vietnam and KS was born in the USA. The independent codings were discussed between the two coders to determine the final coding. Discrepancies were resolved through consensus. Codes were compared between groups when possible (students *v*. parents).

## Results

Two themes reflecting the TAM constructs of perceived usefulness and perceived ease of use were identified. Subcategories included sharing problems with others and information (perceived usefulness); and accessibility, convenience, and low salience of mental health websites (perceived ease of use). Two additional themes, internet addiction and format, also emerged from coding.

### Perceived usefulness

Focus group participants thought an online intervention for mental health would be useful in primarily two ways: sharing problems with others and learning information about mental illness.

### Sharing problems with others

In general, youth feel the internet is a good medium to share their emotions with friends and strangers. As one student said, ‘There should be mental health web pages in which people can talk and share problems.’ Regarding a website, another student suggested, ‘It should have an ‘Add Friends’ option so that patients can connect with each other to share information and their experiences.’ A few parents also shared this sentiment. For example, one parent said, ‘When students surf this web page, it will be easy for them to exchange information comfortably, naturally, and confidently.’

In addition to sharing feelings, students and parents felt that online peers could help students solve their problems. Specifically, students mentioned friends and people with mental illness as sources of help: ‘The ways in which the internet is helpful for stress include sharing with friends and finding answers online.’ Another student said, ‘If there is such a web page, it will help Mai and other people like her solve their problems and overcome depression.’ Anonymity was rarely mentioned by focus group participants and was only a concern for a few participants. As one student stated, ‘For those who have psychological problem, they can access the web for problem solving and sharing without being known.’ In contrast, another student stated, ‘The web is only for treatment and exercises for patients, not the exchange information between patients and other people.’

### Information

Many participants felt the primary role of the internet is to find information about mental illness. Participants believed this knowledge could be leveraged to prevent or manage mental illness. One student said, ‘The web helps patients and their family members find information about mental illness and treatment. Family members can help the patient with exercises.’ Likewise, a parent stated, ‘Having a web page for mental health will be useful because the web page will provide students with knowledge as well as symptoms to prevent or to seek treatment.’ Parents suggested that the website for mental health should help students’ access health care providers. One parent said, ‘Students can know address, telephone and treatment (of a provider) on this web page.’

Notably, some expressed doubts about the information provided on existing web resources for mental health. One student felt that current websites were not suited for youth as illustrated by this quote: ‘I have visited websites to search information, but I have never seen any web pages referring to students’ psychology. There are only some web pages for those over 25.’

### Perceived ease of use

The perceived ease of use was best categorized into three aspects: accessibility, convenience, and low salience of mental health websites. These subthemes related to the efforts users would expend using an online intervention.

### Accessibility

Many stated that accessing the internet was easy. Among high school and university students, 50 and 60% of students had internet at home, respectively. Parents said 70% of their children use the internet. One university student said, ‘Internet access is simple, anybody at any age can do it with a network computer.’ Parents added that students can surf the Internet using computers, iPads, and telephones. However, not everyone has access to the internet as emphasized by one parent: ‘Children living in mountainous areas have few opportunities to surf the Internet. If these children had better living conditions, they could access the internet…’ Similarly, a student said, ‘If students don't have internet at home, then where?’

### Convenience

Participants thought that internet-delivered care would save users time and money. One parent stated, ‘When students surf this web page, they can look for information on their problem without traveling, which can save money, time and travel expenses.’ One student suggested that the internet can be used for follow-up treatment: ‘For patients who had treatment at hospital, they can do exercises at home.’

Still, logistical concerns regarding the proper setting to use an online intervention were also prominent. For example, one college student stressed, ‘(A website is) not convenient for students in a dormitory because students live in a shared room, which is not private.’ Similarly, some students felt that an internet cafe would not be ideal because it is crowded, costly, and not private. Another student offered the home as an ideal setting: ‘The most suitable place to complete an online intervention is at home, because outside will be crowded and noisy. There is a quiet atmosphere at home, patients feel comfortable and can more easily express themselves.’

### Low salience of mental health websites

The low salience of web-based intervention for mental illness was commonly mentioned by participants. As one student said, ‘Students and pupils often access websites on reproductive and sexual health. They won't access mental health websites due to lack of knowledge about mental health.’ The vast majority of participants agreed that these websites on mental health are not well advertised.

Participants had multiple solutions to increase the salience of mental health websites. They recommended using online, newspaper and television promotions, linking interventions to youth websites, and letting educators know about the website. Parents recommended training sessions in the community. Also, participants recommended piloting online services among patients who come to the psychiatric hospital.

One university student stated:

“In my opinion, few students will access this web page as it is not well known. To promote online services, it needs to published and advertised on the web to increase the curiosity in the community. We can work with secondary schools, high schools, and universities to promote the website. This way students can introduce the website to other people.”

### Internet addiction

Internet addiction was identified as an important concern for parents and may affect comfort with an internet-based intervention for youth. Most of parents’ concern centered on online gaming. Parents believe gaming has negative effects on health and academic performance. One parent remarked: ‘Nowadays, surfing the Internet to look for information is easy. It depends why they look for information. But most students spend most of free time playing online games after school.’ Another parent said, ‘If children become addicted to games, they forget to eat and sleep, which affects their health and schoolwork.’ A few parents expressed worry that games can lead to mischievous behavior, including disobedience and even crime. As one parent complained, ‘Children are tempted to play games, but sometimes when they do not have any money, they can commit a crime.’ Remarkably, no students mentioned concerns about internet or gaming addiction.

### Format

There was no clear consensus on who would recommend an internet intervention to youth. Students were more comfortable with family and friends introducing the intervention. One female student remarked, ‘My friends will do that. I will feel safe and more confident if my friends recommend it to me.’ Another student added, ‘I will feel more comfortable if recommended by my brothers, sisters or relatives.’ Students also thought doctors could introduce the website. Though some parents agreed with students that friends should introduce the website, most felt authority figures should refer students to the website. Specifically, parents mentioned psychiatrists, psychologists, teachers, and parents.

Participants expressed some ideas on the content of the website. Most students were reluctant to spend more than 30 min at a time on a mental health website. As one student put, ‘Thirty minutes. If it takes longer, I will feel tired, bored and feel like I am taking a test.’ Parents had more input on the features of the website. They felt the website should give real success stories on overcoming depression. The following examples illustrate this:

*Have lively images through pictures, and stories*.

*To have this web page, we need a detailed plan, accurate and practical contents, images, attractive stories, and videos*.

## Discussion

Internet-delivered treatment has the potential to expand mental health treatment for Vietnamese youth. A basic prerequisite is that this form of treatment delivery be embraced by intended users, youth, and those close to them, their parents. To the authors’ knowledge, this study is the first study describing the perceptions of Vietnamese youth and their parents regarding internet-based interventions for mental health. Using the TAM as a basic framework, we identified sharing with others, providing information on mental health, the low salience of mental health websites, internet addiction, and format components as prominent themes.

Participants emphasized that an internet intervention providing psychoeducation would be useful. Participants wanted to know the symptoms and treatment of mental illnesses. This finding is encouraging given that prior studies have shown low levels of knowledge mental health literacy in Vietnam (van der Ham *et al*. 2011). Notably, the content of the program should cater to a younger demographic as some student participants felt that existing websites discussing mental illness were mostly geared toward adults.

Importantly, as youth mentioned, psychoeducation should be available to family members as well. Because of strong Confucian values in Vietnam, parents have a strong influence on the decisions of youth (Tran, 2001). Unfortunately, many Vietnamese parents may not seek help for their children due to lack of knowledge about mental illness and stigma (Doan, 2011). Further, family education about mental illness is not part of the medical model of mental illness treatment in Vietnam (Niemi *et al*. 2010). In focus groups on mental health literacy, we found that parents did not felt well-versed in the psychological development of their children (unpublished), though in the current study parents did not explicitly state how the internet could improve their own psychoeducation. Web-based resources that provide psychoeducation can fill in the knowledge gap for parents. More research is necessary to determine if increased caregiver knowledge will improve the management of youth mental illness and promote help-seeking.

In addition to psychoeducation, many participants expressed a wish to share information and advice with others online. This finding corresponds with a recent study of over 1100 Vietnamese high school students where over 60% wanted to share private mental health problems and seek help online (Nguyen *et al*. 2013). Moreover, 90% of students in that study stated they would visit such a website if it existed. Similarly, in a study exploring the perceptions of Irish adolescents regarding mental health mobile applications (Kenny *et al*. 2016), participants desired that mobile applications allow for interaction with peers to provide support and advice. In addition, another study of an internet intervention for preventing youth depression reported users wanted more interaction with peers (Iloabachie *et al*. 2011). Indeed, worldwide the use the internet to interact with peers about mental health continues to gain popularity (Burns & Slovic, 2012). Because many Vietnamese youth often turn to their friends for help (Phuong *et al*. 2013) and social networking sites are very popular in Vietnam, online social support will likely be an appealing delivery format for youth. Notably, only a few high-quality studies of peer support for mental health have been conducted and results are mixed (Ali *et al*. 2015). As these studies were all conducted in Western high-income countries, it remains whether this approach will be an effective treatment modality in the Vietnamese context.

Participants expressed a number of advantages of web-based treatment. As commonly reported in other studies (Horgan & Sweeney, 2010; Andersson *et al*. 2014), participants felt the internet would save time and money. Given the centralization of mental health treatment in psychiatric hospitals and ongoing challenges to integrated care in primary care clinic (Lee *et al*. 2015), internet interventions can increase access to care by decreasing or eliminating travel time. Psychotropic medications are the main treatment modality for mental disorders in Vietnam as psychotherapy is limited to wealthy individuals living in large cities. By using the internet, evidence-based psychotherapies could reach many more youth.

However, internet access is not ubiquitous. While 50–60% of students in the study had the internet at home, participants recognize the internet is not easily accessed in remote areas. In addition, finding a comfortable place to use an online program appears to be a challenge. There were concerns about privacy for youth living in dormitories or accessing the program via internet cafes. Providing standalone internet service centers near school and universities as one student suggested may be a viable solution. A number of participants favored the home as a setting to use a web-based program.

Even if the internet is available in a comfortable setting and an internet intervention was developed, youth and parents must be informed that internet interventions exist. Many participants mentioned that websites providing information on mental health were difficult to find. Studies demonstrate lack of knowledge of internet-based treatment is a barrier (Gun *et al*. 2011; Carper *et al*. 2013). Increasing knowledge of internet treatment increases intentions to use computerized programs (Mitchell & Gordon, 2007). One recommendation from students was to partner with the Youth Union, the largest socio-political group in Vietnam, to increase the salience of internet interventions. The Youth Union advocates for youth civic and socioeconomic development and is widely represented in university campuses throughout the country. Therefore, the union has the capacity to inform many youth of online treatment. Alternatively, online advertising has been successfully used to recruit people with mental disorders for research studies (Batterham, 2014), including internet interventions (Morgan *et al*. 2013). Advertising on Facebook, which is the most popular social networking site in Vietnam, is feasible and has been done in multiple studies (Pedersen & Kurz, 2016). These solutions may have merit as youth in this study preferred referral to websites by peers and relatives.

While most disadvantages reported by participants align with previous studies (Ebert *et al*. 2015), one unique concern in this study was internet addiction. Parents strongly felt that youth overuse the internet, particularly for gaming purposes. Internet addiction has been widely reported in Asia. Although less empirical work has been done in Vietnam, one recent government study found that over 70% of high school students play online video games (CNN, 2010). Consequently, the Vietnamese government has imposed restrictions on internet cafes and added taxes to curtail youth online gaming. It is unclear whether parents in this study were concerned that internet interventions would serve as a gateway to use of the internet for other purposes, whether they were concerned about the total time spent on the internet, or both. To address these concerns, an internet intervention could screen users for internet addiction. Alternatively, incorporating a component addressing addiction may be helpful in this context.

A number of limitations of this study are worth considering. First, the participants discussed an intervention that did not exist. Participants’ conception of an internet intervention likely differs and affected their perceptions. However, our approach was to focus on a model of acceptance rather than a specific intervention. Future research could determine whether the TAM model and other themes identified in this study correspond with use of an actual internet intervention in Vietnam. Further, future studies can present website mock-ups to participants to provide more consistency. Another limitation is the youth sample was all students. Because education in Vietnam is compulsory through elementary school, a significant number of youth do not attend high school. Consequently, the current sample characteristics limit the transferability to all youth. Also, the sample size of 20 youth and 20 parents of youth, while not large, generated a number of themes. Our work is exploratory in nature and corresponding quantitative data is justified. Finally, we did not explicit ask participants about mobile phone-based interventions. Mobile devices, including smartphones, are widely used in LMICs. Delineation between internet-based and mobile-based (i.e. applications) interventions in future research is warranted.

## Conclusion

Despite the demonstrated effectiveness of internet interventions for mental health, there is a need to assess acceptability in new contexts. To our knowledge, this is the first qualitative study of web-based mental health treatment in Vietnam. Internet interventions for youth mental health could be a promising way to increase access to care. Results suggest interventions including psychoeducation and social networking will be well received. However, barriers need to be considered to successfully implement an intervention. The findings in the study inform future work on internet interventions for mental health in Vietnam. More broadly, the findings offer important insights for the development of digital health interventions for mental health in other settings. This research indicates a theory of acceptance such at the TAM provides a useful organizing structure to assess willingness to use digital health technologies for mental health. Researchers should incorporate the aspects of acceptance, including usefulness and ease of use to guide design and facilitate use of future interventions.
